# Smartphone-Based Grading and Rehabilitation in Patients With Facial Palsy Using Computer Vision: Prospective Validation Study

**DOI:** 10.2196/81963

**Published:** 2026-05-25

**Authors:** Franz-Tassilo Müller-Graff, Fabian Essig, Maximilian U Friedrich, Kathrin Hoika, Stephan Hackenberg, Kristen Rak, Johannes Taeger

**Affiliations:** 1 Department of Oto-Rhino-Laryngology, Head and Neck Surgery, University Hospital Wuerzburg, Josef-Schneider-Strasse 11, Wuerzburg, 97080, Germany, 49 093120121485; 2 Department of Neurology, University Hospital Wuerzburg, Wuerzburg, Germany; 3 Department of Neurology, University Hospital Ulm, Ulm, Germany

**Keywords:** Digital Facial Index, facial palsy, facial neuromuscular retraining, facial diagnostics, truedepth camera, smartphone sensors, mHealth, usability, telerehabilitation, computer vision, mobile health

## Abstract

**Background:**

Peripheral facial palsy causes significant functional and psychosocial impairments, requiring precise assessment and patient engagement for effective rehabilitation. However, conventional clinician-graded scales (eg, House-Brackmann Scale, Sunnybrook Facial Grading System, and Stennert Index) are subjective and prone to interobserver variability, limiting their reliability for tracking recovery. Smartphone-based computer vision solutions offer objective, standardized facial movement grading, and interactive home-based training to improve adherence and outcomes.

**Objective:**

This pilot study evaluated a novel iOS smartphone app (Apple Inc.) for facial palsy management. The app uses the iPhone TrueDepth 3D camera and on-device computer vision to compute a Digital Facial Index (DFI) for objective facial movement analysis, and provides guided neuromuscular facial exercises with real-time biofeedback. The study aimed to validate DFI against standard clinical grading scales and assess patient-reported outcomes and usability.

**Methods:**

A 4-week single-arm pilot included 21 patients with unilateral facial palsy. Participants used the app at home for daily facial exercises and periodic self-assessments with DFI. Clinicians, blinded to DFI, rated facial function from standardized video exams at baseline and 4 weeks using the House-Brackmann Scale, the Sunnybrook Facial Grading System, and the Stennert Index. DFI concurrent validity was evaluated via correlation with these clinician scores. Patient-reported outcomes included pre- and postintervention Facial Disability Index (FDI) physical and social scores, the System Usability Scale, and a poststudy user feedback questionnaire.

**Results:**

During the study period, strong correlations were observed between DFI and conventional clinical scores. FDI physical and social showed significant functional improvement. Mean System Usability Scale was 88.3 (SD 15.4), indicating excellent usability, and participants reported high satisfaction, preferring the app over traditional paper-based exercises.

**Conclusions:**

The app’s DFI provided objective facial function grading that correlated well with standard clinical scales. Patients’ FDI scores improved significantly over 4 weeks. High usability and patient preference support the app’s feasibility for home-based rehabilitation. This digital approach is promising for facial palsy management, and controlled studies are needed to confirm efficacy and improve long-term engagement.

## Introduction

Facial nerve palsy (FNP) is a common neurological disorder affecting approximately 30 per 100,000 individuals annually, often leading to significant functional and aesthetic impairments [1,2]. Difficulties with facial expressions, speech articulation, and ingestion severely impact quality of life [3,4]. The treatment of FNP encompasses pharmacological, surgical, and rehabilitative strategies [5]. Among these, facial neuromuscular retraining is a widely recommended noninvasive approach aimed at restoring facial motor function [6,7]. Traditionally, such training relies on printed exercise instructions, requiring patients to perform facial exercises in front of a mirror as a biofeedback mechanism. Everyday clinical experience shows that adherence is generally low, and the evidence base for its effectiveness remains variable [8]. Albeit more efficacious, supervised therapy by specialized therapists is limited by cost and availability, especially in rural areas and therefore underused [9,10].

Effective FNP management requires tools for precise diagnostic assessment, structured rehabilitation training, and disease monitoring. The current standard of care is semiquantitative, clinician-administered visual scales such as the House-Brackmann Scale (HBS) [11] and the Sunnybrook Facial Grading System (SFGS) [12-14]. Other established scales include the Facial Nerve Grading System 2.0 [15], movement, rest, secondary defects, and subjective scoring grading system described by de Ru et al [16], Yanagihara Scale [17], or the Stennert Index (SI) [18]. Despite their widespread use, these approaches are limited by time-intensive scoring and are affected by clinimetric limitations such as subjectivity and interobserver variability [19], prompting innovative solutions.

Efforts to improve objectivity in FNP assessment encompass standardized photo and video documentation as well as semiautomated software solutions [20], which often require specialized and costly hardware, again restricting their widespread clinical adoption [19]. Digital tools such as eFACE (a clinician-graded electronic facial paralysis assessment; Banks et al [21]) support FNP quantification, but are limited by their reliance on manual user input [21].

In recent years, digital medicine approaches have revolutionized health care by leveraging consumer technologies and artificial intelligence (AI) algorithms to enhance diagnostics and disease monitoring [22,23]. In particular, the rapid evolution of smartphone sensors—such as high-resolution cameras and Light Detection and Ranging–based depth-sensing—has opened new possibilities for medical applications [24-28]. These built-in capabilities, coupled with AI methods such as computer vision (CV), enable precise motion tracking, real-time biometric analysis, and automated assessments, reducing the need for specialized equipment. This advancement is particularly valuable in fields requiring vision-based analyses, such as facial nerve assessment and rehabilitation, and might therefore provide an ideal solution that would provide time-efficient, user-friendly, and reproducible assessments, minimizing interobserver variability while maintaining diagnostic accuracy [23]. Indeed, previous pilot studies by our group demonstrated the feasibility of objective FNP assessment as well as app-supported facial rehabilitation using the advanced facial recognition and tracking capabilities afforded by Apple’s TrueDepth camera technology [29,30]. However, the clinical utility of these functionalities remained undetermined.

Here, we evaluate a novel smartphone app combining objective FNP grading and structured rehabilitation training in a clinical setting. The app leverages the iPhone’s TrueDepth camera and on-device CV algorithms to generate a Digital Facial Index (DFI), enabling rapid and reproducible facial movement assessment. Additionally, it features a neuromuscular training program with real-time biofeedback and educational video content to enhance patient engagement and guide proper exercise execution. This solution addresses key limitations in FNP assessment and rehabilitation, offering a highly accessible, scalable, and objective tool.

## Methods

### App Interface

**Figure 1.**
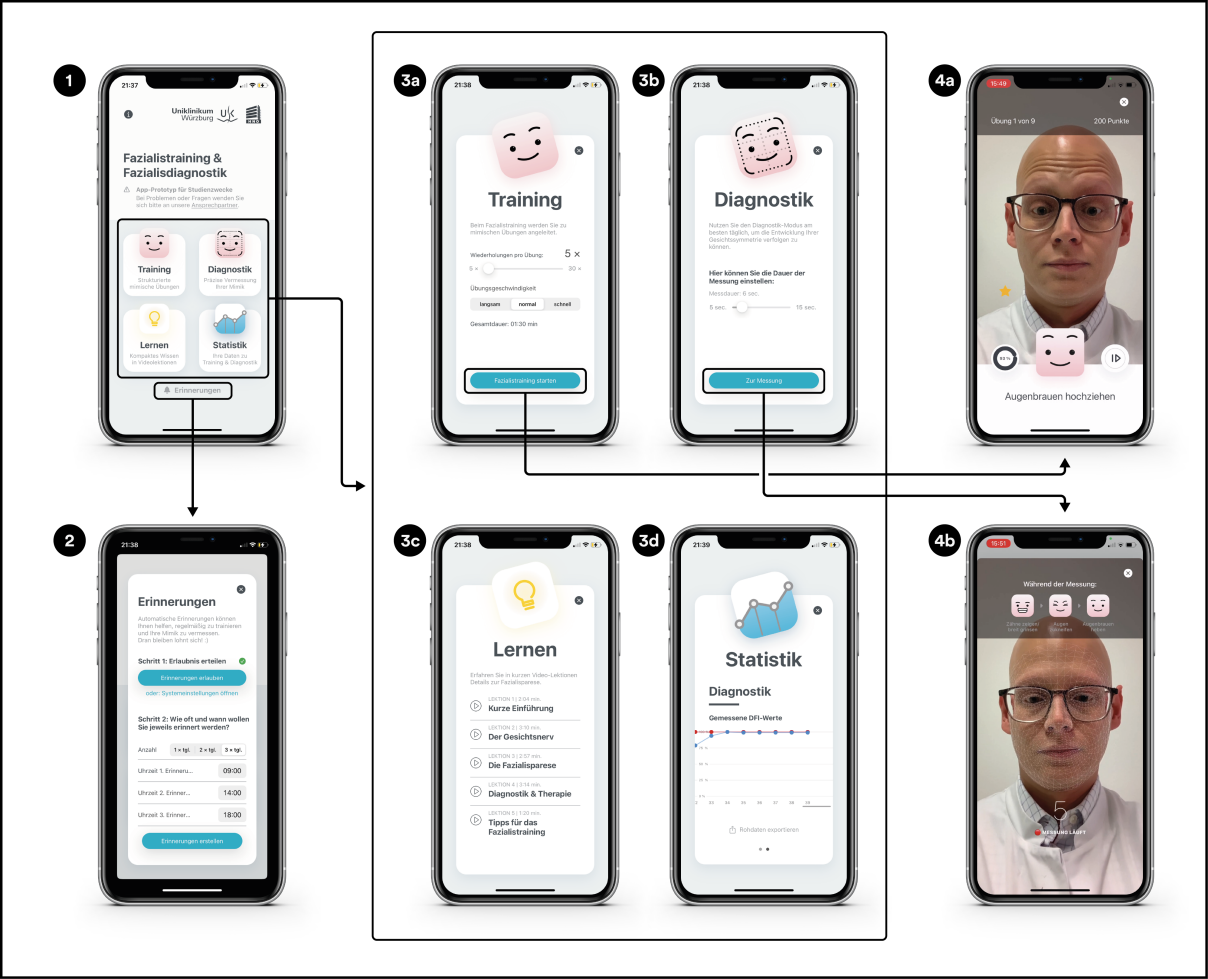
Overview of the study app interface (German version). (1) Home screen with buttons for the 4 main components: facial training (“Training”), diagnostics (“Diagnostik”), learning (“Lernen”), and statistics (“Statistik”), as well as the reminder function. (2) Reminder screen with up to 3 daily reminders. (3a/3b) Setting screens for facial training and facial diagnosis. (3c) Learning screen with 5 video lessons on the subject of facial palsy. (4a) Facial neuromuscular retraining with real-time biofeedback using animated visual cues. (4b) Facial nerve diagnostics with a specification of 3 grimaces, which the user must perform within the previously set time window (in 3b). The app interface is shown in its original German-language version. Written informed consent for the use of the image was obtained from the individual shown.

The app’s diagnostic and training capabilities have been extensively described in the literature [29,30]. In summary, in addition to its core functions, including a facial training program with real-time biofeedback, in which users view a live camera-based reflection of their own face, and facial diagnosis, as outlined in the introduction, the app also features an educational component with easy-to-understand videos explaining the clinical pathophysiology of acute facial palsy. The training module is based on a modified facial exercise protocol described by Okreu and Beckers [31] and comprises 9 standardized facial movements, including eyebrow elevation, eye closure, cheek inflation, lip pursing, lateral mouth movements to the right and left, wide mouth opening, elevation of the mouth corners, and showing the teeth. Exercises are performed in front of the smartphone’s front-facing camera and are supported by real-time visual feedback. Instead of conventional paper-based instructions, exercise execution is guided by animated visual cues and accompanying text instructions. The number of repetitions and training pace can be individually adjusted, and a percentage-based feedback score indicates the accuracy of movement execution. In the current version, the training module is static and provides the same standardized, movement-based facial exercises to all users, independent of DFI values. Users can monitor their individual progress in managing facial paralysis through statistical data and set daily training reminders. An overview of the study app interface is presented in Figure 1.

### App Development

As in the preliminary work, a native app architecture under iOS (Apple Inc.) was selected [29]. This approach allowed for the efficient transfer of existing code directly into the study app, enabling seamless integration with new modules and ensuring full compatibility with the Face ID technology underlying the DFI measurement. The use of on-device computer algorithms via Apple’s ARKit enabled real-time facial analysis without requiring data to leave the device. Common graphic and animation tools, such as the Adobe Creative Suite (Photoshop, Illustrator, and After Effects; Adobe Inc.) and Sketch (Bohemian Coding), were used to design the user interface. The app was developed using the Swift programming language within the XCode development environment (Apple Inc.). Open-source code libraries were integrated via the CocoaPods (Eloy Durán and Fabio Pelosin) dependency manager [32], including “Hero” for general view controller transitions [33], “ScrollableGraphView” for displaying graphs in the statistics function [34], and “Lottie” for vector-based animations used in the animated smileys of the training function [35].

### Grading of FNP

The facial grading tool of the app objectively quantifies facial movements, generating a result known as the DFI. The DFI is calculated based on region-specific facial movement amplitudes derived from 3D depth data captured by the smartphone’s TrueDepth camera during standardized facial expressions. Movements of the forehead, eye region, and mouth are quantified separately, with the affected side normalized relative to the contralateral side. These values are combined into a weighted composite score (forehead 10%, eye region 40%, and mouth region 50%) to generate the final DFI expressed as a percentage. The DFI is expressed on a scale from 0% to 100%, where 0% represents complete paresis and 100% indicates normal, intact facial nerve function. Thus, a maximum value of 100% corresponds to fully preserved and normal facial nerve function.

**Figure 2.**
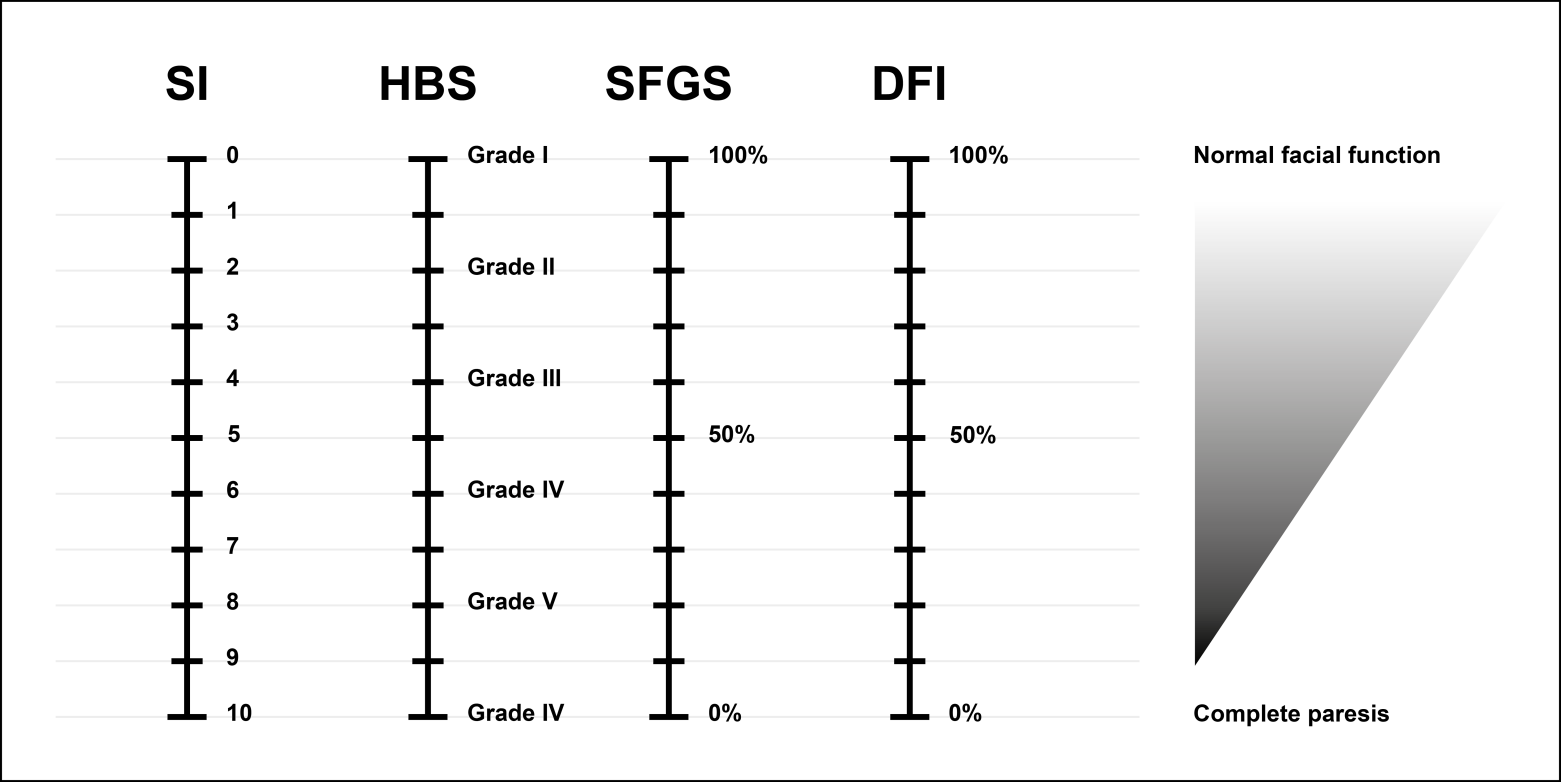
Conversion scheme for the 4 different scores: Stennert Index, House-Brackmann Scale, Sunnybrook Facial Grading System, and Digital Facial Index. DFI: Digital Facial Index; HBS: House-Brackmann Scale; SFGS: Sunnybrook Facial Grading System; SI: Stennert Index.

To compare the DFI with clinically established scoring systems, the SI, HBS, and SFGS were assessed by 3 experienced raters (2 ENT specialists and 1 dental doctoral candidate) using standardized facial videos. Raters were blinded to the DFI results, patient identity, and each other’s assessments. The SI is a historically established grading system commonly used in German-speaking clinical practice and uses inverse scoring, with lower scores indicating better facial nerve function. In relation to these conventional clinical scores commonly used to evaluate facial function, the DFI corresponds directly to the SFGS (0%‐100%) [12] and inversely to the SI (0‐10) [18] and HBS (grade I-VI) [11]. For example, a DFI of 60% is equivalent to an SI of 4, an HBS grade of III, and an SFGS score of 60%. To enhance comparability, all scores were standardized so that 100% reflects fully intact facial function. A conversion chart aligning the various clinical scores (SI, HBS, SFGS, and DFI) is provided in Figure 2.

### Subjective User Assessment

The Facial Disability Index (FDI) is a disease-specific, self-reported questionnaire designed to assess the physical and psychosocial disability associated with facial neuromuscular disorders [36]. It consists of 2 subscales: physical function, which evaluates difficulties in essential activities such as eating, drinking, speaking, and oral hygiene, and social and well-being function, which assesses the emotional and social impact of facial dysfunction, including social withdrawal, irritability, and psychological distress. The FDI has been validated as a reliable tool for measuring patient-reported disability and treatment outcomes, providing a more comprehensive assessment than general health-related quality of life measures [36]. The exact items of the FDI can be found in the Results section.

The System Usability Scale (SUS) is a widely used, standardized questionnaire designed to assess the overall usability of a system [37]. It consists of 10 Likert scale items that evaluate aspects such as ease of use, complexity, consistency, and the need for technical support. The SUS provides a single composite score ranging from 0 to 100, offering a global measure of subjective usability. Originally developed for industrial usability assessments, the SUS is a quick, reliable, and cost-effective tool that has been validated across various domains, making it suitable for comparing different systems and tracking usability improvements over time [37]. A score of 85‐100 is considered “excellent” usability, indicating that the system largely meets user expectations; a score of 70‐84 is classified as “good”; a score of 50‐69 is deemed “acceptable”; and a score below 50 is categorized as “poor,” indicating significant usability issues that require comprehensive improvements [38]. The exact items of the SUS can be found in the Results section.

The study-specific questionnaire is a structured, self-reported assessment tool designed to evaluate user experience and engagement with the facial app used in this study. It consists of 10 Likert scale items, covering aspects such as overall satisfaction, clarity and usefulness of educational videos, ease of use of the diagnostic function, motivation for training, and preference compared to traditional paper-based exercises. Additionally, the questionnaire assesses the effectiveness of features such as progress tracking and reminders in supporting adherence to therapy. An open-ended section allows participants to provide qualitative feedback and suggestions for improvement, making the questionnaire a comprehensive tool for assessing user acceptance and potential optimizations of the app. The exact items of the study-specific questionnaire can be found in the Results section.

### Study Design

The study app was made available to the participants either on loaned iPhones (XR) or on their own compatible devices.

Patients were eligible for inclusion if they had facial palsy of any etiology, provided that a routine diagnostic evaluation had been conducted in the ENT clinic or neurology department of the University Hospital of Würzburg. Only patients in the acute stage of facial palsy, defined as symptom onset within a maximum of 7 days prior to study inclusion, were enrolled. Additional inclusion criteria required participants to be at least 18 years of age and in a clinical condition that permitted study participation, meaning they were awake, cooperative, and capable of providing informed consent. Exclusion criteria comprised patients whose general health status did not allow for informed consent or study participation. Furthermore, individuals with other diseases or prior surgeries in the head and neck region, unrelated to facial palsy but potentially influencing measurement outcomes, were excluded from the study.

Over the course of the 4-week study period, participants were encouraged to use the app regularly for training and diagnostics. At both the beginning and end of the study, standardized videos were recorded according to the protocol suggested by Schaede et al [39], from which 3 experienced raters assessed 3 clinical scores: SI [18], HBS [11], and SFGS [12]. In addition, the DFI was recorded at these time points, and disease-specific quality of life was assessed using the German version of the FDI [36,40]. At the final appointment, the SUS according to Brooke [37] was used to evaluate the usability of the app, and further questions were collected through a tailored, study-specific questionnaire consisting of 10 items. To assess therapy frequency and duration, usage data were retrieved from the app at the final appointment (the app includes a logbook feature that tracks the respective usage times and periods).

### Statistical Analysis

No a priori power analysis was conducted. Due to the exploratory nature of the study, effect sizes are reported alongside *P* values to aid in interpretation. Prior to the analysis of scores and the FDI, the normality of the data was assessed using the Shapiro-Wilk test. Based on the distributional characteristics of the data, nonparametric or parametric tests were applied where appropriate. To compare the facial scores and the FDI between the beginning and the end of the study, the Wilcoxon matched-pairs signed-rank test was used for nonparametric tests and a paired *t* test was used for parametric tests. Differences were considered statistically significant at a significance level of α=.05.

Both Spearman rank correlations were used to assess the relationship between the newly developed Digital Facial Index (DFI) and existing facial palsy scores (SI, HBS, and SFGS). Spearman correlation was used to evaluate the strength and direction of monotonic relationships, making it suitable for ordinal and nonnormally distributed data.

To evaluate the degree of agreement between the DFI and the other facial palsy scores and to identify potential systematic bias, Bland-Altman plots were generated. These plots are presented with bias (mean difference), SD of the bias, and the 95% limits of agreement (LoA).

Intraclass correlation coefficients (ICCs) were calculated to compare the DFI with the SI, HBS, and SFGS, as well as to assess intrarater variability across 3 repeated measurements within each score. Based on the methodology described by Wirtz [41] and Koo and Li [42], a 2-way mixed model was applied, and average-measure ICC values were reported. ICC was tested for absolute agreement and interpreted according to the classification proposed by Cicchetti [43]: unacceptable (ICC<0.4), fair (0.4≤ ICC<0.6), good (0.6≤ ICC<0.75), and excellent (ICC≥0.75). Furthermore, to analyze the consistency of the 3 repeated measurements within each score, ICC with a 2-way mixed model and single-measure values and the associated Cronbach α were computed. Cronbach α was interpreted based on the following scale: unacceptable (α<0.7), fair (0.7≤ α<0.8), good (0.8≤ α<0.9), and excellent (α≥0.9). Only statistically significant ICC and Cronbach α values were considered for interpretation.

Statistical analyses and data visualization were conducted using GraphPad Prism (version 8.4.0; GraphPad Software) and SPSS (version 30.0.0.0; IBM Corp). Data are presented in bar charts, scatter plots, and Bland-Altman plots.

### Ethical Considerations

The prospective study was approved by the Ethics Committee of the University of Würzburg (file number 96‐20-sc), and informed consent was obtained from all participants prior to their inclusion in the research. All participants provided written informed consent prior to inclusion in the study. As facial video recordings were required for the assessment of facial function, the study involved identifiable data. These data were handled confidentially and in accordance with applicable data protection regulations, and access was restricted to authorized study personnel. No financial compensation was provided for participation in this study.

## Results

### Facial Function: Study’s Bookends

A total of 21 patients were included in the study, comprising 11 males (52.4%) and 10 females (47.6%) with a mean age of 40.1 (SD 11.33) years. The underlying causes of unilateral FNP were idiopathic in 10 cases (47.6%) and inflammatory and neoplastic in 7 cases (33.4%), including 6 cases of zoster oticus (28.6%) and 1 case of T2 acoustic neuroma (4.8%). Additionally, 4 cases (19%) were classified as traumatic, comprising 2 cases of FNP following traumatic brain injury with petrous bone fracture (9.5%) and 2 cases of iatrogenic postoperative facial nerve injury (9.5%), which occurred following type III tympanoplasty and parotidectomy. Regarding laterality, facial palsy affected the left side in 11 cases (52.4%) and the right side in 10 cases (47.6%). All participants were included in the acute phase of peripheral facial palsy (≤7 days after symptom onset). Synkinesis, assessed using the synkinesis subscore of the SFGS, was rated as 0 by all 3 blinded raters at both baseline and follow-up.

**Figure 3.**
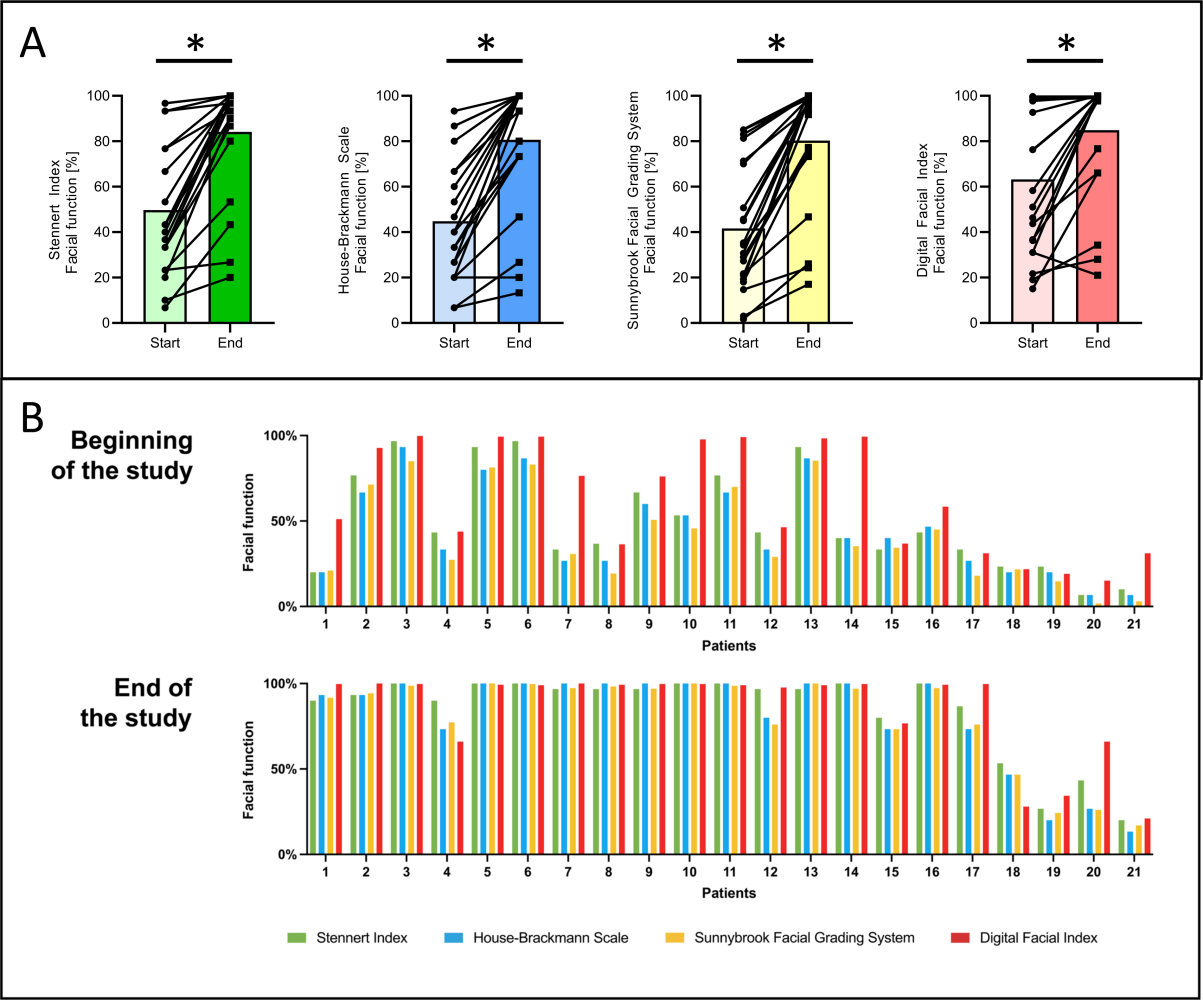
Comparison of facial function at the beginning and end of the study. Line plots illustrating facial function at the beginning and end of the study. A significant improvement is evident across all scoring systems. (B) The Digital Facial Index shows the closest correlation with the House-Brackmann Scale results. A more detailed examination of all participants can be found in (B). At the start of the study, the Digital Facial Index appears to produce overly optimistic results in certain cases compared to the other scoring systems (eg, patient 7). By the end of the study, the results are more consistent, with the Digital Facial Index largely providing an accurate representation of normal facial function. Differences between the different cohorts are indicated as significant,*P<.001 (paired *t* test and Wilcoxon matched-pairs signed-rank test). DFI: Digital Facial Index; HBS: House-Brackmann Scale; SFGS: Sunnybrook Facial Grading System; SI: Stennert Index.

Overall, a statistically significant improvement in facial nerve function was observed across all 4 scoring systems from baseline to study completion. At the onset of the study, the median facial nerve function levels were 43.3% (IQR 33-77) for SI, 40% (IQR 27-67) for HBS, 34.3% (IQR 21-70) for SFGS, and 58.3% (IQR 36-98) for DFI. By the end of the study, these values had all significantly improved to 96.7% (IQR 87-100) for SI (*P*<.001), 100% (IQR 73-100) for HBS (*P*<.001), 97% (76-99) for SFGS (*P*<.001), and 99.3% (IQR 77-100) for DFI (*P*<.001; Figure 3A).

Figure 3B

A more detailed examination of individual patient data shows a high degree of convergence between the DFI and established clinical scores, particularly at the end of the study. While some variability was observed at baseline, overall values aligned more closely over time. Figure 3B provides an overview of individual score trajectories, illustrating that changes observed in clinical grading scales are paralleled by corresponding changes in DFI values. These findings support the clinical validity of the DFI and its responsiveness to functional changes over time.

### Facial Grading Scores: Comparative Analysis

**Figure 4.**
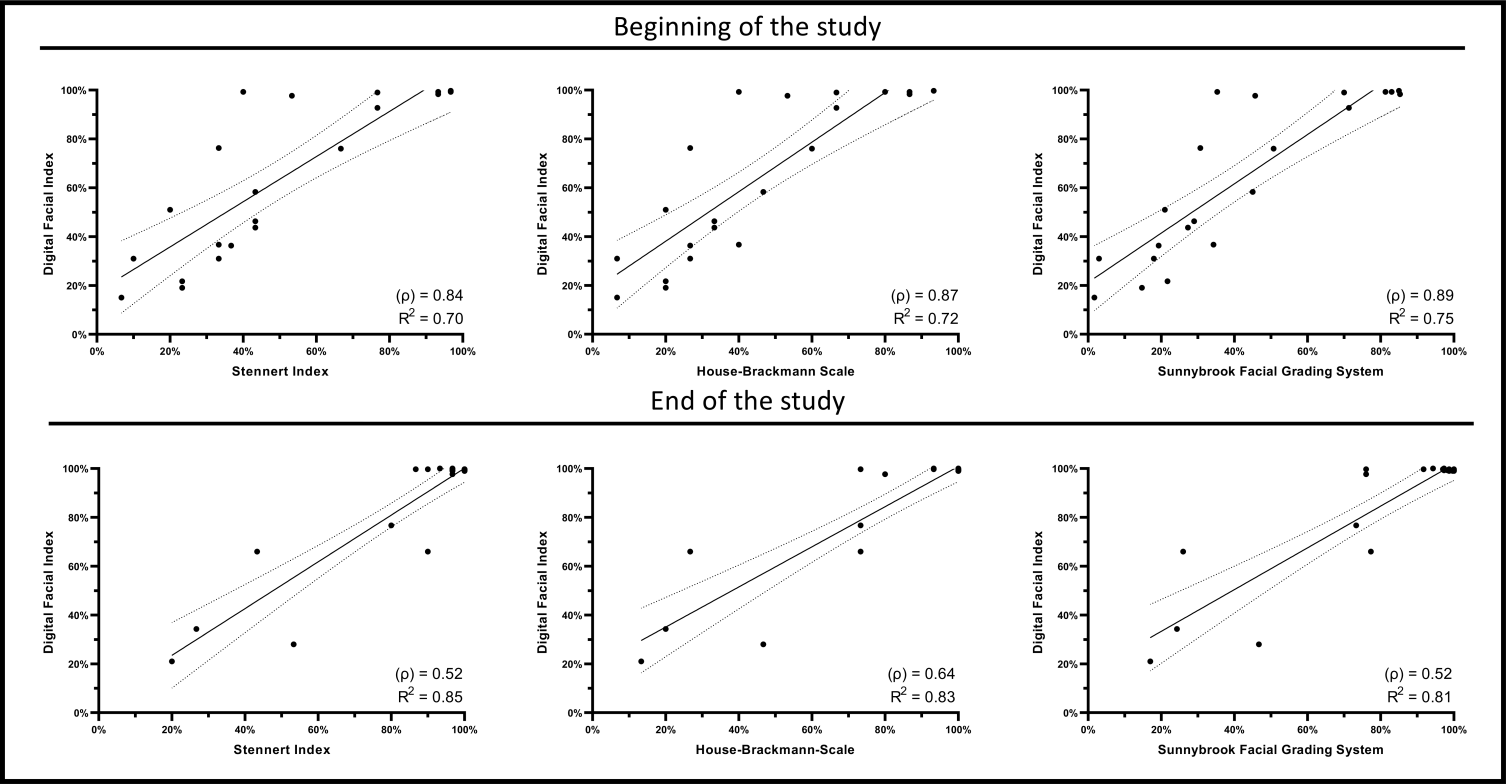
Correlation analysis comparing Digital Facial Index and the other 3 clinical scores shows a good to strong correlation at both the beginning and end of the study. Note that the scores were also adapted here as shown in Figure 2.

In regression analyses, the 3 clinically established scoring systems were compared with the DFI. At the beginning of the study, the Spearman correlation coefficients (ρ) were 0.84 for the SI with the DFI, (ρ)=0.87 for the HBS with the DFI, and (ρ)=0.89 for the SFGS with the DFI, all indicating a strong correlation. By the end of the study, the correlation coefficient for the SI with the DFI decreased to (ρ)=0.52, while the HBS and the SFGS showed correlation coefficients of (ρ)=0.64 and (ρ)=0.52, respectively (Figure 4).

**Figure 5.**
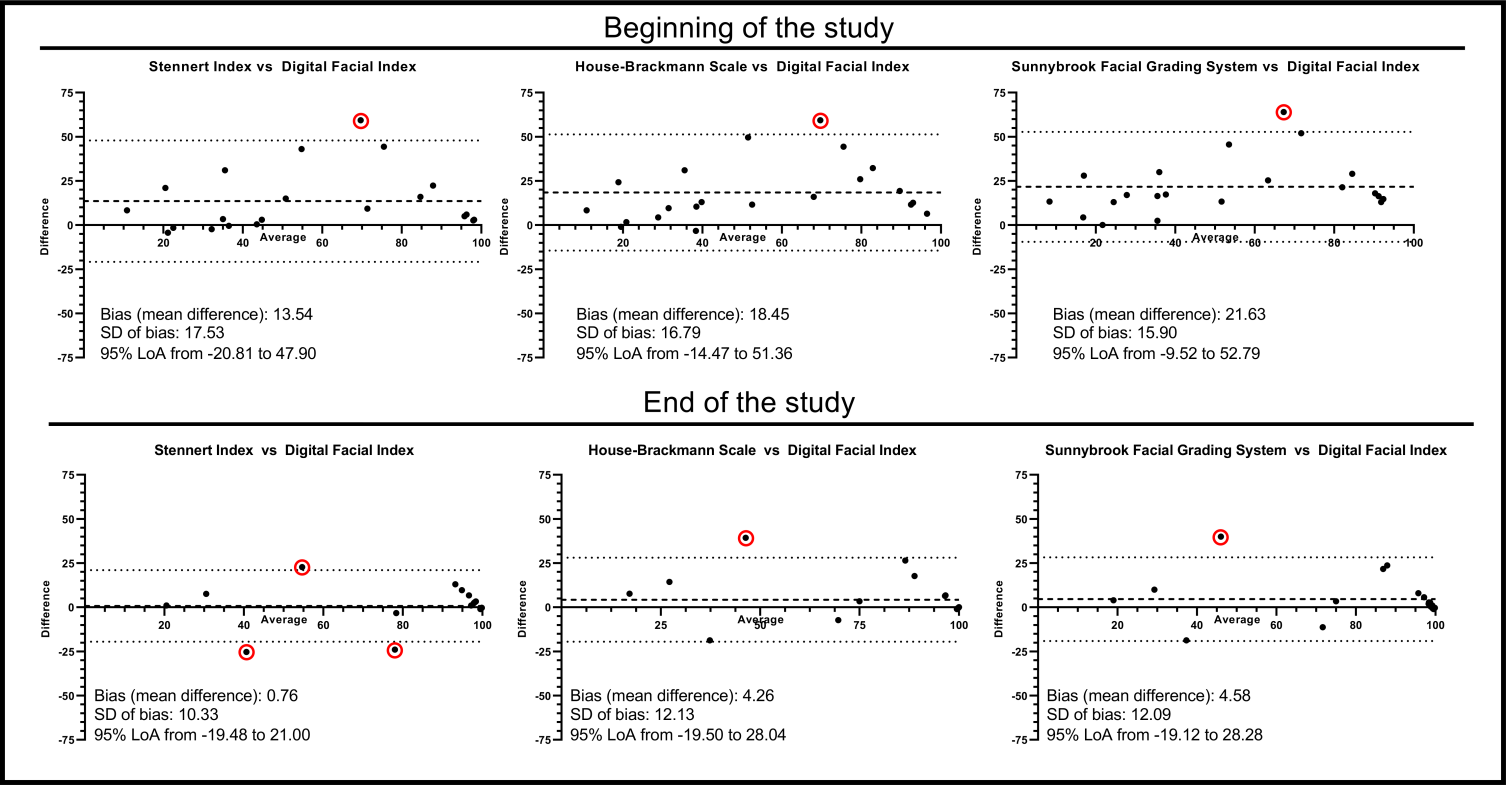
Bland-Altman plots comparing the Digital Facial Index with the Stennert Index, House-Brackmann Scale, and Sunnybrook Facial Grading System at baseline and at the end of the study. LoA: limits of agreement.

Furthermore, Bland-Altman plots were generated to assess the agreement between the clinically established scoring systems and the DFI. These plots confirmed that, at the beginning of the study, the DFI provided systemically more optimistic results regarding facial function compared to the SI (mean difference 13.54, SD 17.53), HBS (mean difference 18.45, SD 16.79), and SFGS (mean difference 21.63, SD 15.90). By the end of the study, this trend was only partially observed (SI: mean difference 0.76, SD 10.3; HBS: mean difference: 4.26, SD 12.13; SFGS: mean difference: 4.58, SD 12.09). Nevertheless, in all plots, the majority of the points fell within the LoA (items out of LoA: SI: 1 beginning, 3 end; HBS: 1 each beginning and end; SFGS: 2 beginning, 2 end), which generally indicates a satisfactory level of agreement between the scores (Figure 5).

To further assess the agreement between the conventional clinical scoring systems and the DFI, both ICC and Cronbach α were calculated. The results were classified as “excellent” (Table 1). Additionally, to evaluate the agreement as well as the internal consistency and reliability of the 3 measurements within the 4 scores (DFI: 3 measurements; SI, HBS, SFGS: 3 raters each providing one rating), both ICC and Cronbach α were calculated. The results were also classified as “excellent” (Table 1).

ICC and CA were used to statistically compare DFI with SI, HBS, and SFGS (average measures) at the beginning and the end of the study. Interpreting the values as described by Cicchetti [43], there are values at both the beginning and the end of the study that can be classified as “excellent.” Furthermore, ICC and CA were used to evaluate agreement within the 3 ratings of the 4 scores (DFI, SI, HBS, and SFGS). As well, all scores were classified as “excellent.”

**Table 1.** Reliability analysis.

	SI^a^ – DFI^b^		HBS^c^ – DFI		SFGS^d^ – DFI		DFI		SI		HBS		SFGS	
	ICC^e^^f^	CA^g^^h^	ICC	CA	ICC	CA	ICC	CA	ICC	CA	ICC	CA	ICC	CA
Between scores														
Beginning of the study	0.828^i^	0.910^i^	0.868^i^	0.910^i^	0.828^i^	0.960^i^	—^j^	—	—	—	—	—	—	—
End of the study	0.946^i^	0.958^i^	0.960^i^	0.958^i^	0.946^i^	0.982^i^	—	—	—	—	—	—	—	—
Within scores														
Beginning of the study	—	—	—	—	—	—	0.908^i^	0.968^i^	0.828^i^	0.950^i^	0.908^i^	0.968^i^	0.828^i^	0.950^i^
End of the study	—	—	—	—	—	—	0.931^i^	0.975^i^	0.914^i^	0.977^i^	0.931^i^	0.975^i^	0.914^i^	0.977^i^

a SI: Stennert Index.

b DFI: Digital Facial Index.

c HBS: House-Brackmann Scale.

d SFGS: Sunnybrook Facial Grading System.

e ICC: intraclass correlation coefficient.

f ICC: excellent (ICC≥0.75); good (0.6≤ICC<0.75); fair (0.4≤ICC<0.6); unacceptable (ICC<0.4).

g CA: Cronbach α.

h CA: excellent (α≥0.9); good (0.8≤α<0.9); fair (0.7≤α<0.8); unacceptable (α<0.7).

i Excellent.

j Not applicable.

### App Usage Frequency

**Figure 6.**
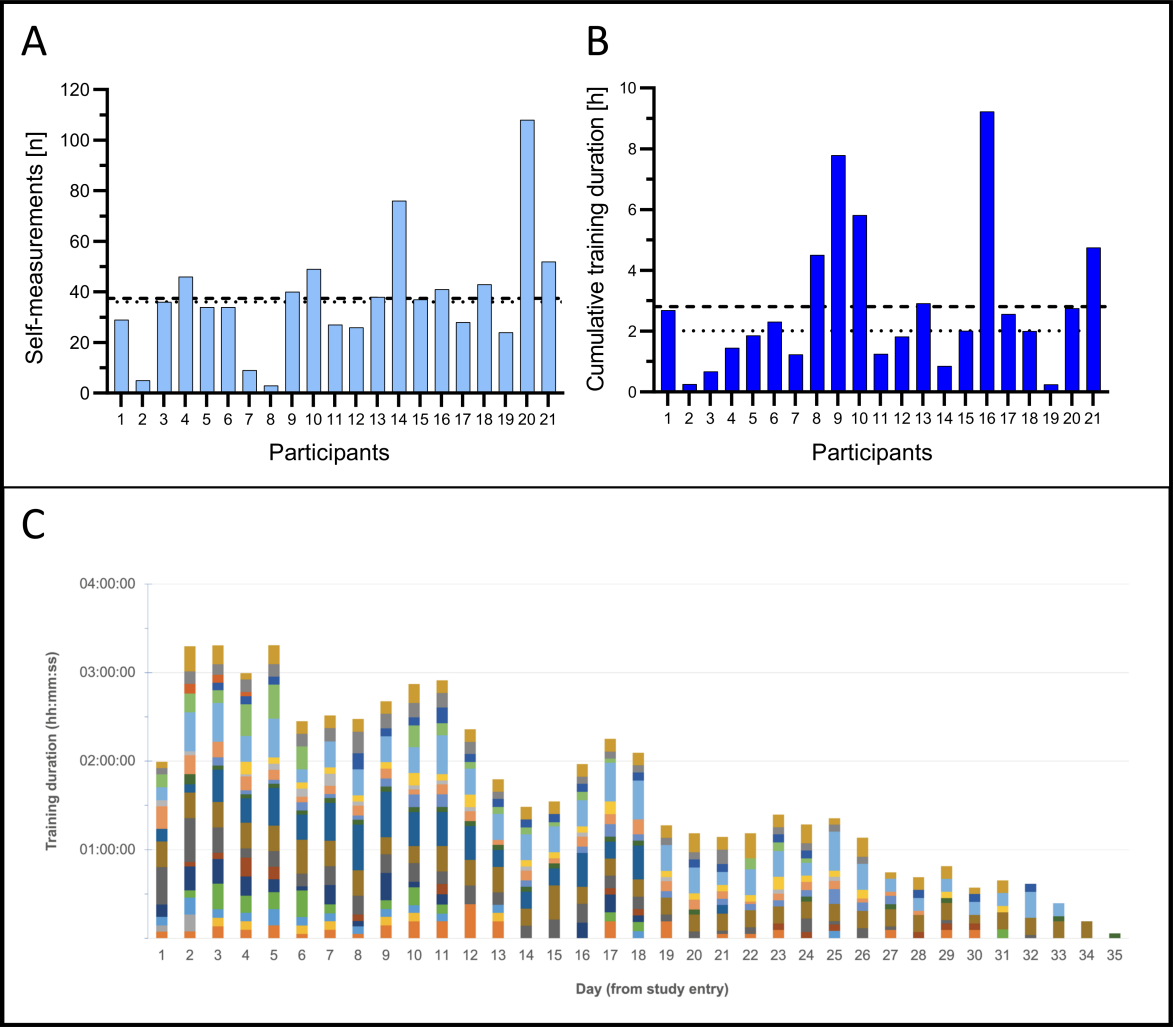
Use of the Digital Facial Index self-measurement and training function within the study app. (A) The Digital Facial Index self-measurements ranged from 3 to 108 (mean 37.4, SD 23.0, dashed line; median 36.0, IQR 27-45, dotted line), shown per participant. (B) Training duration per participant (mean 2 h 48 min, SD 2 h 22 min, dashed line; median 2 h 0 min, IQR 1 h 15 min to 3 h 43 min, dotted line) ranging from 14 min 50 s to 9 h 14 min 33 s. (C) Cumulative facial training time per participant, with each patient represented by a unique color. Due to the individualized scheduling of follow-up appointments, some participants’ involvement extended beyond the initially planned 30 days.

The use of the app was analyzed through a logbook. Regarding the diagnostic function, it was observed that patients used the app in a highly variable manner. The range of usage varied from only 3 applications (case 8) to 108 uses (case 20) of the DFI self-measurements. On average, the participants performed a mean of 37.4 DFI self-measurements (SD 23.0; median 36.0; IQR 27–45) during the study period (Figure 6A).

Regarding the training function of the app, the average mean cumulative usage was 2 hours and 48 minutes (SD 2 h 22 min; median 2 h 0 min, IQR 1 h 15 min to 3 h 43 min) over the 30-day study period (Figure 6B). It should be noted that, due to the individual scheduling of the follow-up appointments, participation sometimes extended beyond the initially planned 30 days up to 35 days from study entry. The cumulative exercise time for all patients is visualized in Figure 6C. A characteristic pattern emerges, resembling a series of progressively lower peaks every sixth day (at approximately days 5, 11, 17, 23, and 29). Between these peaks, training duration exhibits a trough-like decline, creating a wave-like structure. This pattern suggests an initial phase of high engagement, followed by periodic fluctuations and an overall decline in training activity. In the last days of the study, training durations are minimal.

### Subjective User Assessment

The FDI demonstrated a significant improvement in both physical function and social function and well-being among the participants between the start and end of the study. Physical function improved significantly from a mean of 57.1 (SD 13.2) at the beginning of the study to 85.0 (SD 15.2) at the end of the study (*P*<.001). Similarly, the average scores for social function and well-being showed a significant improvement, with the score rising from a mean of 49.3 (SD 16.5) at the start of the study to 83.6 (SD 24.5) at the end (*P*<.001). A detailed list of the questions with the respective means, SDs, and *P* values can be found in Table 2. Correlations between the FDI and other scoring systems ranged from moderate to strong. For the SI, correlations with the FDI increased from *r*=0.41 to *r*=0.61 (physical) and from *r*=0.50 to *r*=0.66 (social). With the HBS, correlations rose from *r*=0.46 to *r*=0.72 (physical) and from *r*=0.67 to *r*=0.74 (social). The SFGS showed increases from *r*=0.53 to *r*=0.74 (physical) and from *r*=0.53 to *r*=0.71 (social). Correlations with the DFI remained stable for physical function (*r*=0.50) and increased slightly for social function (*r*=0.52 to *r*=0.56).

**Table 2.** Evaluation of the Facial Disability Index (according to VanSwearingen and Brach [36] and Volk et al [40]).

Questionnaire items	Facial Disability Index - Item	Beginning of the study, mean (SD)	End of the study, mean (SD)	*P* value
	Physical function			
1	How much difficulty did you have keeping food in your mouth, moving food around in your mouth, or getting food stuck in your cheek while eating?	3.3 (0.8)	4.4 (0.8)	<.001^a^
2	How much difficulty did you have drinking from a cup?	3.1 (0.8)	4.5 (0.7)	<.001^b^
3	How much difficulty did you have saying specific sounds while speaking?	3.8 (0.9)	4.5 (0.9)	<.001^c^
4	How much difficulty did you have with your eye tearing excessively or becoming dry?	3.0 (0.6)	4.2 (0.5)	<.001^b^
5	How much difficulty did you have with brushing your teeth or rinsing your mouth?	3.3 (0.6)	4.4 (0.7)	<.001^b^
	Overall score in %	57.1 (13.2)	85.0 (15.2)	<.001^b^
	Social and well-being function			
6	How much of the time have you felt calm and peaceful?	2.8 (1.0)	4.5 (0.7)	<.001^b^
7	How much of the time did you isolate yourself from people around you?	2.8 (1.0)	4.4 (1.0)	<.001^b^
8	How much of the time did you get irritable toward those around you?	3.3 (1.1)	4.3 (1.1)	<.001^a^
9	How often did you wake up early or wake up several times during your nighttime sleep?	3.3 (1.1)	4.3 (1.2)	<.001^c^
10	How often has your facial function kept you from going out to eat, shop, or participate in family or social activities?	2.7 (0.8)	4.2 (1.3)	<.001^a^
	Overall score in %	49.3 (16.5)	83.6 (24.5)	<.001^b^

a *P*<.001.

b *P*<.0001.

c *P*<.01.

The SUS indicated that the system was generally perceived as highly user-friendly. It should be noted that the SUS score ranges from 0 to 4, with 4 representing the most favorable response, and that negative items (items 2, 4, 6, 8, and 10) are reversed to ensure consistency in interpretation. Positive items, such as “I thought the system was easy to use” and “I would imagine that most people would learn to use this system very quickly,” scored an average of 3.81 (SD 0.51) and 3.62 (SD 0.50), respectively. Negative items, such as “I found the system unnecessarily complex” and “I think that I would need the support of a technical person to be able to use this system” scored 3.57 (SD 0.68) and 3.52 (1.12), suggesting that participants did not perceive these aspects negatively. The overall mean SUS score was 88.33 (SD 15.36; out of 100), which is considered excellent, indicating high user-friendliness and that the system met users’ expectations to a large extent. A detailed list of the questions with the respective means and SDs can be found in Table 3.

**Table 3.** System Usability Score (according to Brooke [37]).

Item	Item end of the study	Positive (P), Negative (N) item	Value, mean (SD)
1	I think that I would like to use this system frequently	P (0‐4)	2.67 (1.28)
2	I found the system unnecessarily complex	N (0‐4 reversed)	3.57 (0.68)
3	I thought the system was easy to use	P (0‐4)	3.81 (0.51)
4	I think that I would need the support of a technical person to be able to use this system	N (0‐4 reversed)	3.52 (1.12)
5	I found the various functions in this system were well integrated	P (0‐4)	3.67 (0.66)
6	I thought there was too much inconsistency in this system	N (0‐4 reversed)	3.67 (0.66)
7	I would imagine that most people would learn to use this system very quickly	P (0‐4)	3.62 (0.50)
8	I found the system very cumbersome to use	N (0‐4 reversed)	3.57 (0.68)
9	I felt very confident using the system	P (0‐4)	3.67 (0.73)
10	I needed to learn a lot of things before I could get going with this system	N (0‐4 reversed)	3.57 (0.68)
	SUS^a^ (sum of all points x 2.5)	Range: 0‐40	88.33 (15.36)

a SUS: System Usability Scale.

A study-specific questionnaire was used to assess participants’ perceptions of the facial nerve rehabilitation app (Taeger) and its various functions. Overall, the results indicate a predominantly positive evaluation of the app, with a mean score of 4.1 (SD 0.9). Participants particularly appreciated the clarity and usefulness of the instructional videos (mean 4.9, SD 0.3) and the explanation of training exercises (mean 4.9, SD 0.3). The app was generally well-received, with high enjoyment ratings (mean 4.5, SD 0.9) and straightforward usability of the diagnostic function (mean 4.4, SD 0.7).

However, user engagement with certain features was more variable. While the diagnostic function provided motivation for regular training (mean 3.9, SD 1.1), the training results (mean 3.2, SD 1.2), statistics function (mean 3.1, SD 1.4), and reminder function (mean 3.1, SD 1.3) were rated lower. Despite these aspects, the majority of participants preferred using the app over the paper-based exercise log (mean 3.9, SD 1.3) and supported the app as a standard supplementary therapy for facial palsy (mean 4.8, SD 0.5). A detailed list of the questions with the respective means, medians, and SDs can be found in Table 4.

**Table 4.** Study-specific questionnaire. Likert scale with 1 (strongly disagree) to 5 (strongly agree).

Item end of the study	Value, mean (SD)
I enjoyed using the facial rehabilitation app.	4.5 (0.9)
The instructional videos were easy to understand and helpful.	4.9 (0.3)
Using the diagnostic function was straightforward.	4.4 (0.7)
The diagnostic function motivated me to use the training regularly.	3.9 (1.1)
The training exercises were clearly explained.	4.9 (0.3)
The training results encouraged me to continue with further training sessions.	3.2 (1.2)
I regularly used the statistics function.	3.1 (1.4)
The reminder function helped me adhere consistently to my training sessions.	3.1 (1.3)
I prefer using the facial rehabilitation app over the paper-based exercise log.	3.9 (1.3)
The facial rehabilitation app should be offered as a standard supplementary therapy option for facial palsy.	4.8 (0.5)
Overall score (mean)	4.1 (0.9)

## Discussion

### Facial Function

Digital apps are increasingly integral to modern medicine, leveraging smartphone sensors for diverse clinical applications. From heart rate monitoring [44-46] to retinal imaging [47-49] and sleep tracking [50-52], smartphones have demonstrated their potential across various medical specialties. In this context, the iPhone’s TrueDepth camera has been explored for assessing facial nerve function, as first described by Taeger et al [29,30]. This study prospectively evaluated a corresponding app-based diagnostic and training approach in a clinical cohort. Our findings highlight 3 main strengths of the DFI: first, it demonstrated high convergent validity, with strong correlations to established clinical grading systems such as the HBS, SFGS, and SI, confirming its consistency with clinical standards. Second, the DFI may offer higher sensitivity in detecting subtle improvements over time, potentially capturing changes that conventional ordinal scales may overlook. Third, user feedback was positive, indicating good usability and patient acceptance of the app-based approach.

A cohort of 21 patients with unilateral facial palsy was assessed under standardized conditions using the newly developed DFI, alongside 3 clinician-graded scoring systems recommended by the German guideline for facial palsy management [5]: the SI, the HBS, and the SFGS. Our findings indicate a strong correlation between the DFI and conventional grading methods over a 4-week follow-up, suggesting that this app-based tool offers a standardized, digital approach to facial function assessment. By integrating objective motion analysis with smartphone accessibility, the DFI may enhance diagnostic consistency and facilitate long-term patient monitoring.

Across all scoring systems, facial nerve function improved significantly over 4 weeks. The initially higher values measured with the DFI suggest that it tends to underestimate the severity of facial palsy. Therefore, correction factors or an optimized analysis algorithm may need to be applied in the future. However, given the exploratory nature of this pilot study and the limited sample size, no statistical correction for the observed DFI overestimation was applied, as such adjustments would be unstable and potentially misleading. Robust calibration approaches will require larger, stratified studies with multiple measurement time points and in-depth raw data analysis. By study completion, these discrepancies diminished, with all measures converging toward near-complete recovery, consistent with the natural course of idiopathic facial palsy, where most cases resolve within 3 weeks [53,54]. As facial function improves across participants, variability between scoring methods naturally decreases. Given that all patients were examined in the acute phase of facial palsy, the absence of synkinesis is in line with the expected disease course and indicates that the present findings primarily apply to early-stage facial nerve dysfunction.

Regression analyses showed strong initial correlations between the DFI and traditional grading systems (ρ=0.84‐0.89), which decreased by study end (ρ=0.52‐0.64). This decline likely reflects reduced variance as patients approached full recovery, resulting in ceiling effects where categorical scales fail to capture subtle residual asymmetries. Similar patterns were described by Katsumi et al [55], who reported reduced score variability in late recovery stages when comparing 3D imaging-based assessments to the HBS [55]. In contrast, the DFI appears more sensitive to minor deficits, potentially indicating greater granularity or capturing distinct aspects of facial nerve recovery. Bland-Altman analyses confirmed an initial bias toward higher DFI scores, which diminished over time, supporting overall agreement with conventional assessments. Reliability metrics, including ICC and Cronbach α, demonstrated excellent reproducibility across all tools, underscoring the robustness of the DFI. However, the divergence in late-stage correlations raises the question of whether the DFI measures distinct recovery aspects or simply offers greater granularity. Further studies with larger, more diverse populations are needed to validate its clinical utility.

It is not uncommon for different clinical grading scales to yield varying results, as each system emphasizes distinct aspects of facial nerve dysfunction. The HBS, for instance, a 6-grade categorical system, is widely used for its simplicity but exhibits substantial grading shifts when converted to other scales. In contrast, the SFGS provides finer gradations, improving precision but requiring more detailed assessment. Studies have demonstrated the superior reliability of SFGS over HBS [56-58], highlighting the advantages of nuanced grading.

While increased complexity enhances accuracy, it can also hinder routine clinical use due to time constraints [56]. The DFI, using a 0%‐100% scale within a smartphone-based app, streamlines data collection and reduces clinician workload. A key innovation is the integration of on-device CV algorithms that automatically analyze facial movements captured via the smartphone camera. This enables high-fidelity, real-time tracking without manual input, improving accessibility and efficiency. Compared to eFACE [21,59], the current digital standard for facial nerve assessment, the DFI offers a key advantage: fully automated video-based data acquisition. While eFACE is highly reliable, it requires manual input, limiting practicality in time-sensitive environments. Independent recording of the DFI by the patient is possible after just a few trials within a time window of only 5 seconds. Automated tools such as the Facogram (O’Reilly et al [60]) have been shown to enhance grading consistency and reduce subjectivity [60,61]. Similar to the Facogram, the DFI aims to improve reproducibility and minimize interobserver variability, offering a real-time, app-based solution for longitudinal monitoring.

### App Usage Frequency

The analysis of app usage frequency provided insights into user engagement with the diagnostic and training functions of the digital facial app. DFI self-measurements varied widely among participants, ranging from 3 to 108 applications. This broad distribution suggests that while some patients actively integrated the app into their routine, others engaged only sporadically. The mean of 37.4 self-measurements (SD 23.0) indicates regular use, reflecting a general willingness to incorporate digital self-assessments into rehabilitation. Similar trends have been observed in other mobile health (mHealth) interventions, where engagement variability is influenced by individual motivation, technological familiarity, and perceived benefit [62-65].

The training function showed a cumulative usage of 2 hours 48 minutes, with wave-like engagement patterns similar to those seen in digital rehab tools [66,67]. While declining use over time could reflect waning motivation [68], it may also indicate functional recovery and reduced perceived need, consistent with prior findings [69-71]. Although efficacy cannot be assessed without controlled studies, usage trends suggest good acceptance of app-based training. Due to the short observation period and the heterogeneous usage patterns, app usage data were interpreted descriptively and not used to infer training efficacy.

Despite these fluctuations, usage patterns support the feasibility of app-based rehabilitation for facial nerve dysfunction. The DFI’s integration into a mobile platform facilitated frequent, flexible self-assessment—an advantage over clinic-based evaluations. High variability in engagement suggests that while some patients adopt digital tools seamlessly, others require structured guidance or reminders [72,73]. Research indicates that engagement in digital health interventions can be improved through gamification, personalized reminders, or real-time feedback [74,75].

Similar variability has been observed in other mHealth apps for facial nerve rehabilitation. Machetanz et al [76] reported differing engagement levels with the FACEsemper app (Machetanz et al [76]), with higher adherence among patients with more pronounced functional deficits. Their study highlights the role of perceived need in sustaining engagement and suggests that automated reminders and tailored exercises could enhance adherence.

Incorporating individualized training plans, manual assistance prompts, or region-specific exercise recommendations may further optimize adherence and therapeutic efficacy. Future studies should explore whether such refinements sustain engagement and improve functional recovery compared to conventional rehabilitation approaches.

### Subjective User Assessment

The subjective evaluation of the app-based rehabilitation approach for FNP showed promising results, with significant improvements in disease-specific quality of life (FDI). The increase in physical function (mean 57.1, SD 13.2 to mean 85.0, SD 15.2; *P*<.001) and social well-being (mean 49.3, SD 16.5 to mean 83.6, SD 24.5; *P*<.001) suggests that digital rehabilitation tools may support motor recovery and broader psychosocial well-being, consistent with previous mHealth studies [77,78]. However, these improvements likely reflect the natural recovery trajectory of FNP rather than a direct effect of the app. While the intervention facilitated structured rehabilitation, its precise impact on quality of life remains unclear. Future controlled studies are necessary to differentiate app-related benefits from spontaneous recovery. Nonetheless, these findings highlight the role of digital tools in rehabilitation and the psychosocial management of FNP. Currently, no studies have directly examined the FDI in digital rehabilitation contexts, such as mHealth solutions. However, integrating the FDI into digital programs, including the app used in this study, could provide valuable insights into their effectiveness.

Usability is critical for long-term adoption. The app achieved a SUS score of 88.3, exceeding the commonly reported average of 68 for digital health apps [37,79], which is also confirmed for digital health apps [80]. This suggests that patients found the app intuitive, well-integrated, and easy to navigate, with minimal technological barriers. Our SUS results align with findings by Machetanz et al [76], who reported a SUS score of 89.6 for a mobile facial grading solution.

Despite positive feedback on core functionalities, secondary features such as statistical feedback and reminders received lower ratings (mean 3.1, SD 1.4 and mean 3.1, SD 1.3, respectively), indicating a need for refinement. Research suggests that engagement in digital health interventions can be enhanced through gamification, personalized progress tracking, and adaptive feedback [81,82]. Future iterations should integrate such elements to optimize user motivation and adherence. A strong preference for the app over traditional paper-based exercise logs underscores a broader shift toward digital health solutions. The ability to perform self-assessments using the DFI and engage in structured rehabilitation without continuous clinical supervision represents a significant advancement in accessibility and patient autonomy in facial palsy management.

This study has several limitations. First, the single-arm design without a control group and the short observation period preclude conclusions regarding therapeutic efficacy. Improvements in facial function are likely dominated by spontaneous recovery, particularly in idiopathic peripheral facial palsy. Second, the sample size was small and heterogeneous with respect to etiology and recovery stage, limiting generalizability. Third, the DFI tended to overestimate facial function in early recovery stages, and no correction factors were applied in this exploratory pilot study.

### Limitations

This study has several limitations. The single-arm design without a control group and the short observation period preclude conclusions regarding therapeutic efficacy, as improvements in facial function are likely influenced by spontaneous recovery, particularly in acute peripheral facial palsy. The small and etiologically heterogeneous sample limits generalizability. The DFI tended to overestimate facial function in early recovery stages, and no correction factors were applied in this exploratory pilot study. Furthermore, the app does not currently detect synkinetic movements or monitor muscle overuse, and its educational content does not address synkinesis; therefore, applicability to patients with chronic facial palsy or established synkinesis cannot be inferred. Accordingly, the present findings should be interpreted as evidence of feasibility, usability, and concurrent validity rather than clinical effectiveness. Future controlled studies with larger cohorts and longer follow-up are required.

### Conclusion

This study highlights the feasibility of an app-based approach for FNP assessment and rehabilitation. The DFI strongly correlated with conventional grading systems, offering a standardized and objective evaluation method. High usability (SUS=88.3) supports its integration into clinical practice, though engagement varied, emphasizing the need for personalized adherence strategies. Future developments such as gamification, adaptive feedback, and AI-driven personalization could further optimize long-term adherence and therapeutic efficacy.

## Abbreviations

AI: artificial intelligence
CV: computer vision
DFI: Digital Facial Index
FDI: Facial Disability Index
FNP: facial nerve palsy
HBS: House-Brackmann Scale
ICC: intraclass correlation coefficient
LoA: limits of agreement
mHealth: mobile health
SFGS: Sunnybrook Facial Gradings Scale
SI: Stennert Index
SUS: System Usability Scale
